# Landscape of internal N7-methylguanosine of long non-coding RNA modifications in resistant acute myeloid leukemia

**DOI:** 10.1186/s12864-023-09526-8

**Published:** 2023-07-27

**Authors:** Jingyi Han, Qinqin Liu, Yao Zhou, Dong Li, Ran Wang

**Affiliations:** 1 Department of Thoracic Surgery, Qilu Hospital of Shandong University, Jinan, Shandong China; 2 Department of Pediatrics, Qilu Hospital of Shandong University, Jinan, Shandong China; 3 Department of Hematology, Qilu Hospital of Shandong University, Jinan, Shandong China

**Keywords:** N7-methylguanosine, Long non-coding RNAs, Acute myeloid leukemia

## Abstract

**Background:**

Growing evidence indicates that RNA methylation plays a fundamental role in epigenetic regulation, which is associated with the tumorigenesis and drug resistance. Among them, acute myeloid leukemia (AML), as the top acute leukemia for adults, is a deadly disease threatening human health. Although N7-methylguanosine (m7G) has been identified as an important regulatory modification, its distribution has still remained elusive.

**Methods:**

The present study aimed to explore the long non-coding RNA (lncRNA) functional profile of m7G in AML and drug-resistant AML cells. The transcriptome-wide m7G methylation of lncRNA was analyzed in AML and drug-resistant AML cells. RNA MeRIP-seq was performed to identify m7G peaks on lncRNA and differences in m7G distribution between AML and drug-resistant AML cells. The Gene Ontology (GO) and the Kyoto Encyclopedia of Genes and Genomes (KEGG) pathway enrichment analyses were conducted to predict the possible roles and m7G-associated pathway.

**Results:**

Using m7G peak sequencing, it was found that a sequence motif was necessary for m7G methylation in drug-resistant AML lncRNA. Unsupervised hierarchical cluster analysis confirmed that lncRNA m7G methylation occurred more frequently in drug-resistant AML cells than in AML cells. RNA sequencing demonstrated that more genes were upregulated by methylation in drug-resistant AML cells, while methylation downregulated more genes in AML cells. The GO and KEGG pathway enrichment analyses revealed that genes having a significant correlation with m7G sites in lncRNA were involved in drug-resistant AML signaling pathways.

**Conclusion:**

Significant differences in the levels and patterns of m7G methylation between drug-resistant AML cells and AML cells were revealed. Furthermore, the cellular functions potentially influenced by m7G in drug-resistant AML cells were predicted, providing evidence implicating m7G-mediated lncRNA epigenetic regulation in the progression of drug resistance in AML. These findings highlight the involvement of m7G in the development of drug resistance in AML.

## Introduction

Acute myeloid leukemia (AML) is a type of blood cancer that originates in the bone marrow and is characterized by the uncontrolled growth of myeloid cells. Drug resistance in AML remains a significant challenge to effective treatment, with several patients relapsing after initial therapy. Understanding the mechanisms underlying drug resistance is therefore an essential step towards developing novel therapies [1]. Emerging evidence suggests that RNA methylation, particularly m7G methylation, of long non-coding RNAs (lncRNAs) plays a critical role in the development of drug resistance in AML. Alterations in RNA methylation patterns, including aberrant m7G methylation of lncRNAs, have been implicated in the acquisition and maintenance of drug resistance in AML [2].

Dysregulated m7G methylation of specific lncRNAs has been associated with altered gene expression patterns, dysfunctions in cellular signaling pathways, and the development of drug-resistant phenotypes in AML cells. Aberrant m7G methylation of lncRNAs has been found to be associated with the modulation of drug efflux pumps, drug metabolism pathways, DNA repair mechanisms, and anti-apoptotic processes, contributing to the resistance of AML cells to therapeutic interventions [3]. Targeting dysregulated m7G methylation of drug-resistant AML-associated lncRNAs presents a potential strategy for overcoming treatment resistance and improving patient outcomes. Comprehensive profiling of RNA methylation patterns, including specific analysis of m7G methylation in lncRNAs, may enable the identification of novel biomarkers and therapeutic targets for combating drug resistance in AML [4]. Integration of RNA methylation data, including the assessment of m7G methylation in lncRNAs, with clinical and genomic information can enhance our understanding of the mechanisms driving drug resistance in AML and guide the development of personalized treatment strategies.

As a post-transcriptional modification, RNA methylation plays a fundamental role in epigenetic regulation. To date, over 100 RNA methylations have been reported [5]. Examples are m7G (7-methyl guanosine), m5C (5-methylcytosine), m1A (N1-methyladenosine), m6Am (2-O-dimethyladenosine), and m6A (N6-methyladenosine). During epigenetic regulation, RNA methylation influences various bio-processes, including RNA splicing, translation, nuclear export, stability, DNA damage repair, initiation of miRNA biogenesis and immunogenicity, thereby affecting cellular differentiation, embryonic development, spermatogenesis, sex determination, learning and memory, immune response and occurrence and development of cancer [6]. For instance, SRY-box transcription factor 2 modification by m6A in glioblastoma is positively correlated with radiation resistance and maintenance of cells similar to glioma stem via METTL3, and m1A in tRNA drives liver tumorigenesis by regulating cholesterol metabolism[7, 8].

Notably, m7G is a widely recognized RNA modification on mRNA caps and inside rRNAs, tRNAs and mRNAs[9]. Despite its abundance, m7G is highly difficult to be investigated by using standard sequencing-based technologies owing to high stability in neutral aqueous solutions and neutrality to Watson-Crick base pair of m7G in RNA, without affecting reverse transcription. In virtue of advances in techniques such as high-throughput sequencing, m7G modifications in RNA species with low contents of m7G, including lncRNAs and mRNA, can be identified and quantified. Previous studies have reported functions and distribution of m7G in different classes of RNA. Nevertheless, understanding of distribution over the transcriptome and prevalence of m7G in lncRNA remains at its infancy [10–12].

LncRNAs are RNA molecules with over 200 nucleotides but no or limited protein-coding potential. Indeed, LncRNAs serve as key gene expression regulators in multiple pathological and physiological processes, including tumorigenesis, cell differentiation, and inflammation [13]. Nowadays, lncRNAs have attracted great attention in post-transcriptional regulation, as well as epigenetic modification. Specifically, lncRNAs interact with RNAs, proteins and chromatins in cytoplasm/nucleus, which is associated with multiple diseases (e.g., diverse types of cancer) [14–16]. For instance, lncRNA MEG3 contributes to drug resistance in AML by positively regulating ALG9 through sponging miR-155[17].

According to the American Cancer Society, around 20,000 cases of AML were diagnosed in 2020in the United States. Despite some breakthroughs in AML diagnosis and treatment over the recent decades, relapsed and refractory nature of AML remains significant owing to its heterogeneity and malignancy [18]. Nevertheless, recent studies revealed a key role of methylation modifications in AML progression, demonstrating it as a novel effective AML therapy [19–22].

In the present study, the influence of m7G methylation on lncRNAs in AML-resistant cells was investigated. Specifically, m7G was globally mapped via RNA MeRIP-seq in AML-resistant and non-resistant cells, while distribution and prevalence in the two cell types and cellular compartments were determined and compared with each other. The results demonstrated that the m7G modification degree was significantly higher in AML-resistant cells than that in non-resistant cells. This could be reflected by intra-cell consistency, as well as inter-cells differences, involving all chromosomes. This study could facilitate the understanding of epigenetic regulation of lncRNAs in AML by m7G, as well as new therapies for AML.

## Materials and methods

### Cell lines and cell culture

HL60/MX2 and HL60 cells were acquired from the American Type Culture Collection and cultured in RPMI-1640 containing 10% thermally deactivated fetal calf serum, 1% (v/v) penicillin streptomycin in a humidified atmosphere containing 5% CO_2_ at 37 °C.

### Development of cDNA libraries

In the present study, m7G peaks were acquired from each sample by using the MeRIP-seq technique. RIP was executed by a commercial service provider. High-quality reads were aligned to the UCSC HG19 by utilizing hisat2 (ver. 2.0.4). Then, fragments per kilobase of exon per million fragments mapped values from transcript-level LncRNAs were obtained by utilizing cuffdiff (ver. 2.2.1) under guidance by the gtf files. Additionally, the P-value and fold-change of the two groups were determined to screen for lncRNAs that were expressed differentially.

### Analysis of sequencing data

Sequencing and visualization of the libraries developed were achieved by using a sequencer (Illumina NovaSeq 6000). The mapping quality is reflected by ‘Q30’: a read above 30 was considered to be reliable. Meanwhile, library mapping was achieved by STAR (ver. 2.5.1b). Sequencing datasets were aligned to UCSC HG19 by utilizing Hisat2 (ver. 2.0.4). Methylation peaks of the two cells were identified by using MACS, while their differences were determined by using DiffReps.

### The GO and KEGG pathway enrichment analyses

The GO and KEGG pathway enrichment analyses were carried out using the DAVID online tool to clarify the properties of the above-mentioned genes. The GO analysis aimed to analyze MFs (molecular functions), CCs (cellular components), and BPs (biological processes) of these genes based on differentially methylated genes. P < 0.05 was set to be the critical level of significant GO terms. Additionally, the KEGG pathway analysis [23–25] was conducted to investigate functions and biological pathways. Similarly, P < 0.05 indicated a significant difference.

## Results

### Identification of LncRNAs related to transcriptome-wide m7G methylation in human AML-resistant cells

**Fig. 1:**
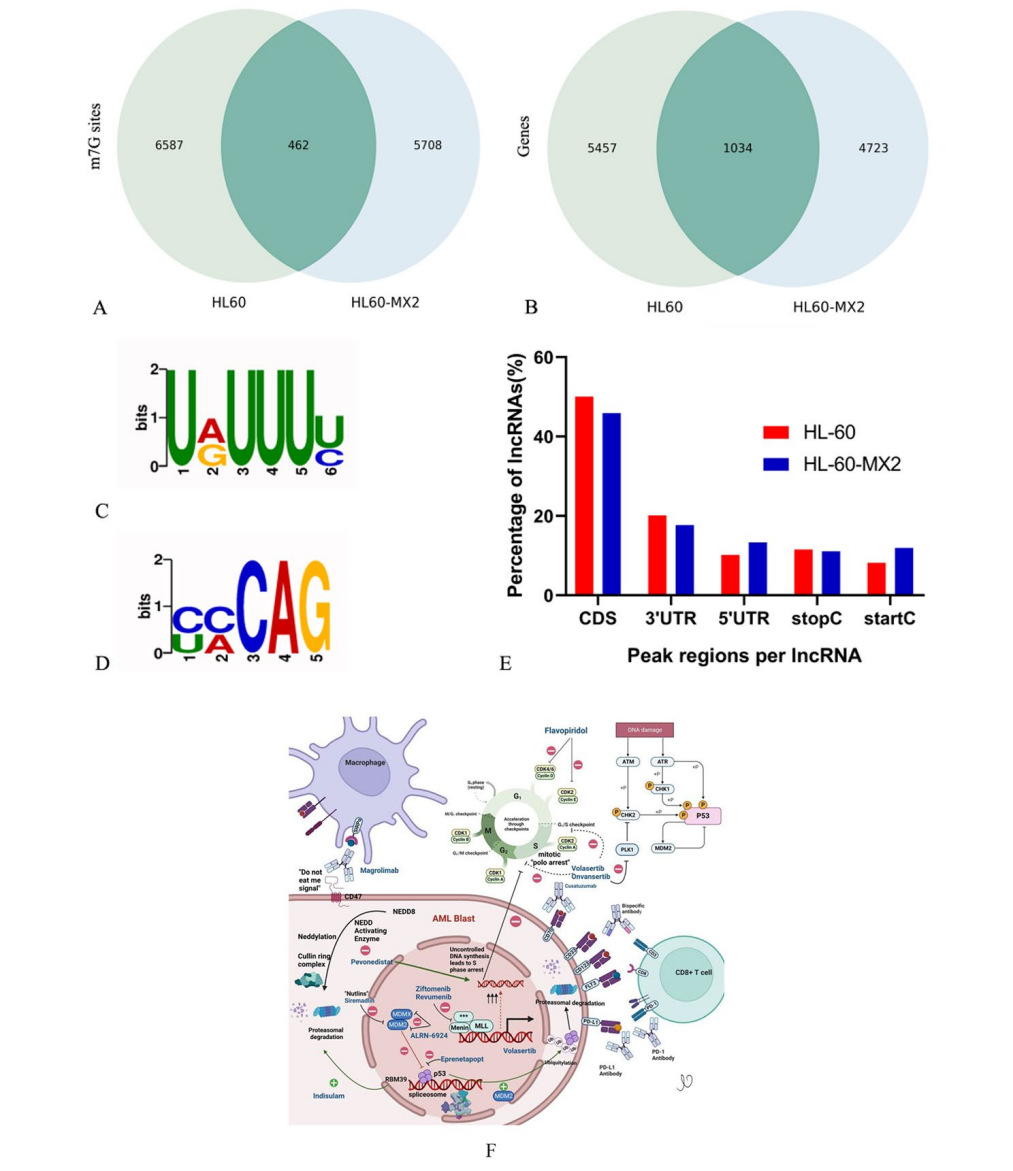
m7G methylation (transcriptome-wide) and role of lncRNAs in the two cells. (**A**) Venn diagram of m7G methylation sites recognized in lncRNAs from the two cells. (**B**) Venn diagram of m7G genes in the two cells. (**C**) The sequence motif of m7G sites in HL-60 cells. (**D**) The sequence motif of m7G sites in MX2/ HL60 cells. (**E**) Percentages of lncRNAs containing different quantities of m7G peaks in the two cells; most lncRNAs contain one m7G peak only. (**F**) A schematic showing the pathways and biological functions affected and how they differ in drug-resistant versus sensitive AML [26]

m7G methylation over transcriptome in the two cells was investigated by RNA MeRIP-seq of lncRNA. As observed, a total of 7,049 and 6,170 m7G peaks were observed in HL-60 cells and HL60/MX2 cells, respectively (Fig. 1a). Up to 6491 and 5,757 annotated genes were mapped in HL-60 and HL60/MX2 cells, respectively (Fig. 1b). Among them, 462 m7G peaks appeared in both HL-60 cells and HL60/MX2 cells. A fold-change cut-off of 2.0 was employed to identify differentially methylated peaks. Additionally, 99% and 84% of sites identified in this study were indeed specific to HL60/MX2 and HL-60 cells, respectively, suggesting that the possibility of m7G methylation is higher in AML-resistant cells than in AML cells.

The sequences of m7G-methylated peaks were investigated by scanning to verify that an m7G motif is present. URUUUY (R = A/G, Y = U/C) was determined to be the most conservative motif in HL-60 cells and its E-value was 1.1e-009 (Fig. 1C). Meanwhile, the most conservative MOTIF for m7G peaks in HL60/MX2 cells was YMCAG (Y = C/U, M = C/A) and its E-value was 9.7e-020 (Fig. 1D), indicating that the MOTIF sequences of m7G methylation exhibited significant difference in the two groups.

Additionally, the density of m7G peaks in lncRNA was determined on the basis of the cell type. The results showed that 50% of lncRNAs comprised of a m7G peak in the CDS region in HL-60 cells, compared to 45.9% of single peak lncRNA in MX2/ HL60 cells. Percentages for the 3’UTR region (20.1% in HL-60 cells vs. 17.7% in HL60/MX2 cells), 5’UTR region (10.2% in HL-60 cells vs. 13.3% in HL60/MX2 cells), stopC (11.5% in HL-60 cells vs. 11.1% in HL60/MX2 cells) and startC (8.2% in HL-60 cells vs. 11.9% in HL60/MX2 cells) were also determined (Fig. 1E). A schematic showing the pathways and biological functions affected and how they differ in drug-resistant versus sensitive AML is presented in Fig. 1F [26].

### Analysis of sources of lncRNAs methylation in the two cells

**Fig. 2:**
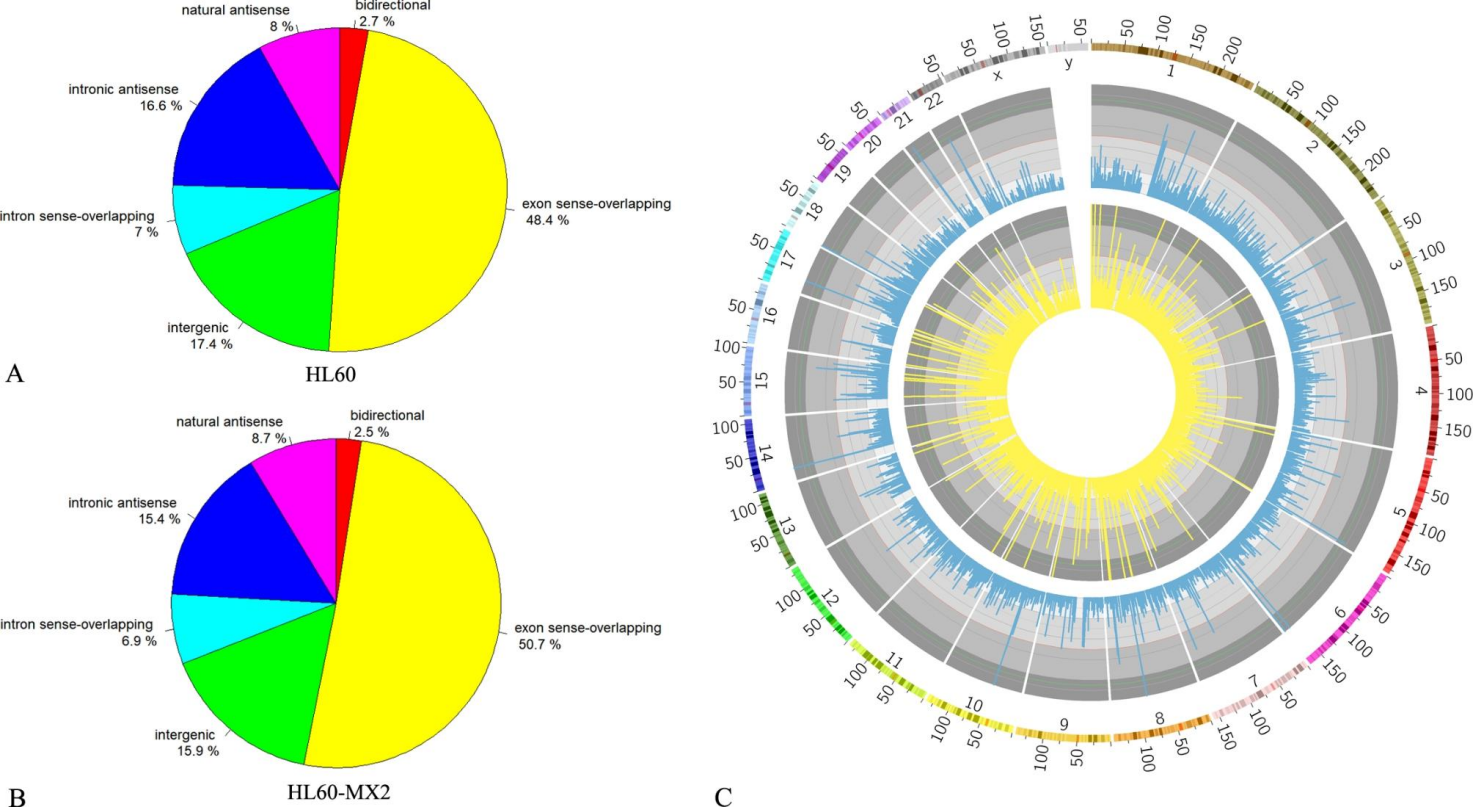
** A** and **B**. Pie chart of the source of methylated lncRNA in the two cells. **C**. Visualized chromosome-level distribution of m7G in lncRNAs in the two cells

The source data of methylated lncRNAs were summarized and a pie chart was plotted (Fig. 2A and B). The results showed that most of the lncRNAs were derived from exon sense overlapping regions, followed by intergenic regions. Compared with HL60 cells, HL60/MX2 cells had fewer methylated lncRNAs derived from intergenic regions, and more methylated lncRNAs from exon sense overlapping regions. Meanwhile, more lncRNAs were derived from exons in HL60/MX2 cells, which may lead to an increase of the number of lncRNAs that can perform silencing and affect cell function. Additionally, the distributions of lncRNA methylation sites on the chromosomes were analyzed by using Circos (Fig. 2C). The results showed that the lncRNA methylation site distribution on each chromosome was different between the two groups. Indeed, the X chromosome and chromosome 2 were the more evident ones. Also, there are various types of leukemia and tumors associated with specific regions on chromosome 2. Compared with autosomes, sex chromosomes in both groups were less methylated.

### Analysis of differential expression of lncRNAs on the basis of m7G methylation

**Fig. 3:**
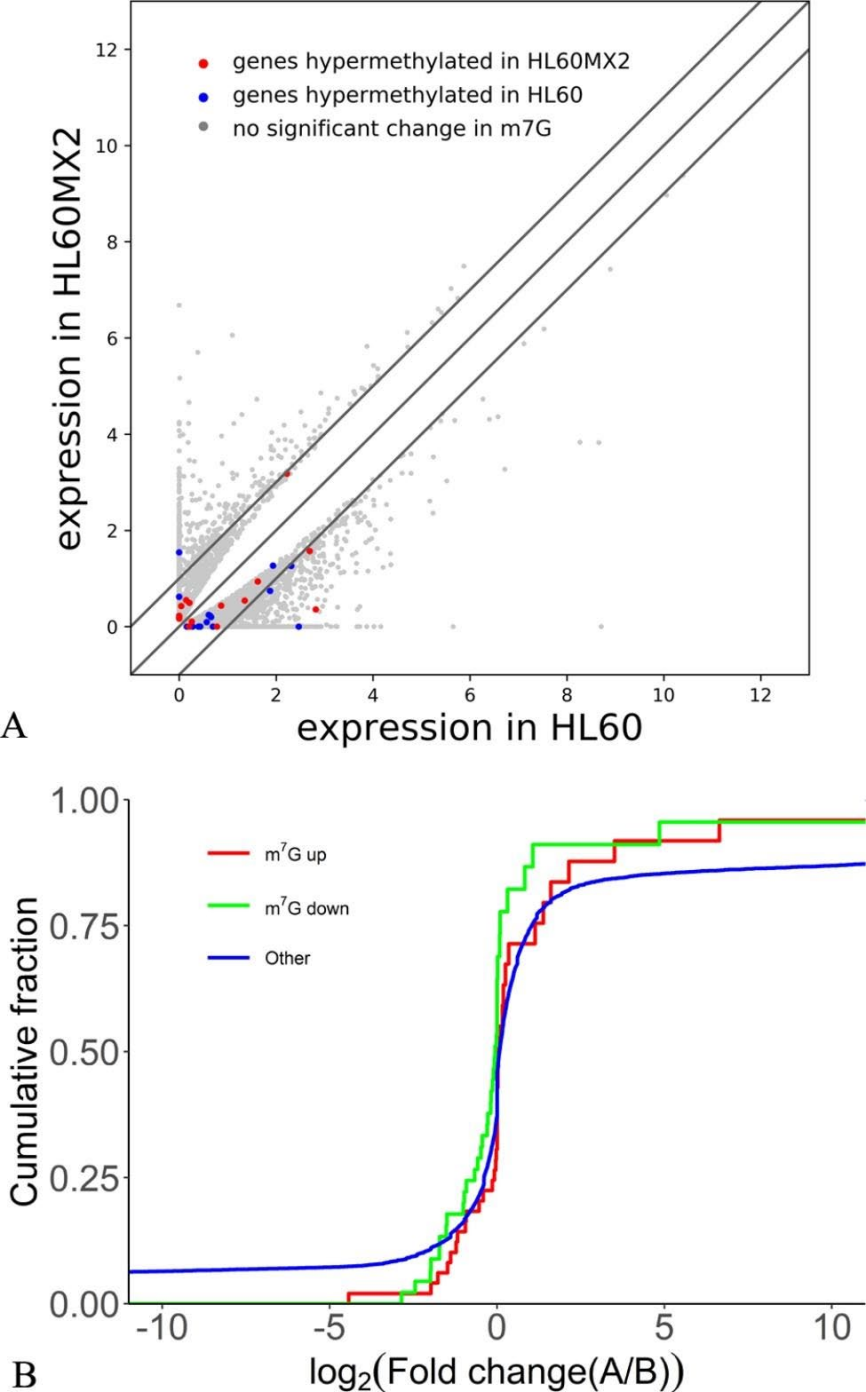
The differentiated expressions of lncRNAs categorized based on m7G methylation. (**A**) Differential expression of lncRNAs in the two cells. (**B**) Cumulative distribution of lncRNA expressions in the two cells for genes with upregulated m7G (red) and downregulated m5C (green); blue denotes others

The expressions of lncRNAs in the two cells were investigated by RNA sequencing according to m7G methylation. It was revealed that 301 lncRNAs were upregulated and exhibited increased expression levels in the HL60/MX2 cells. On the other hand, 355 lncRNAs were downregulated in HL60/MX2 cells compared with HL-60 cells (Fig. 3A). The top 10 downregulated and upregulated lncRNAs are listed in Table 1.

**Table 1:** Top 10 upregulated and downregulated lncRNAs in HL60 and HL60/MX2 cells screened by microarray

chrom	txStart	txEnd	Peak length	Transcript ID	GeneName	Foldchange
Top ten up-methylated peaks						
chr15	41,068,739	41,069,084	345	ENST00000558769	DNAJC17	3509.2
chr1	181,060,281	181,061,020	739	ENST00000606938	RP11-309G3.3	2413.4
chr2	219,114,314	219,114,440	126	ENST00000487321	ARPC2	2164.8
chr19	59,084,283	59,084,740	457	NR_027334	MZF1-AS1	2017.5
chr1	33,977,607	33,978,343	736	ENST00000424879	RP4-580O19.2	1999.1
chr8	8,101,514	8,101,562	48	ENST00000522393	ALG1L13P	1971.5
chr1	12,052,335	12,052,340	5	ENST00000497302	MFN2	1888.6
chr11	70,187,978	70,188,160	182	ENST00000528284	PPFIA1	1879.4
chr14	101,295,370	101,295,620	250	NR_002766	MEG3	1842.5
chr12	57,907,901	57,908,023	122	ENST00000582079	RN7SL312P	1824.1
Top ten down-methylated peaks						
chr15	28,999,954	29,000,320	366	ENST00000512149	WHAMMP2	3161.4
chr17	67,171,759	67,171,922	163	ENST00000519732	ABCA10	2955
chr16	20,610,884	20,611,038	154	ENST00000566754	RP11-143N13.1	2325.1
chr17	45,112,409	45,112,492	83	ENST00000575930	RP11-156P1.3	2064.5
chr18	67,448,539	67,448,658	119	ENST00000577609	DOK6	2042.8
chr12	74,565,501	74,565,610	109	ENST00000551714	RP11-274M17.1	1977.6
chr2	62,327,322	62,327,475	153	ENST00000425966	AC018462.2	1923.3
chr5	17,436,845	17,437,322	477	ENST00000505844	RP11-321E2.3	1890.7
chr6	149,387,311	149,387,390	79	ENST00000466695	UST	1793
chr14	89,885,503	89,885,620	117	NR_036500	FOXN3-AS1	1782.1

The top 10 lncRNAs with statistically different transcription levels of hypermethylated genes are listed in Table 2. Functional abbreviations and names of such hypermethylated genes are summarized in Table 3. These genes may be correlated with AML resistance pathogenesis and shall be further investigated. Additionally, the number of downregulated m7G modified lncRNAs was higher than that of upregulated ones in the HL60/MX2 cells (Fig. 3B). Consequently, m7G may be associated with gene expression in various transcripts of AML resistant cells.

**Table 2:** Top 10 differentially expressed lncRNAs in HL60/MX2 and HL60 cells

chrom	GeneName	Foldchange	log2(fold change)
chr11	MYO7A	443	286.47
chr14	AL589743.1	4.301626417	88.7104
chr7	AC005083.1	627.1	6.63711
chr6	LINC01012	1271.7	3.51199
chr7	PSPH	424.6	2.13992
chr1	AK023809	544	-1.52072
chr15	SRP14-AS1	435.4	-1.72591
chr4	RP11-87F15.2	316	-1.97841
chr1	AB075489	413.7	-1.99104
chr8	KIAA0146	11.24742268	-2.85847

**Table 3:** Functional roles of top 10 differentially expressed lncRNAs

GeneName	Full Name	Gene Type	Function
MYO7A	myosin VIIA	protein coding	a member of the myosin gene family
AL589743.1	None	None	None
AC005083.1	None	None	None
LINC01012	long intergenic non-protein coding RNA 1012	ncRNA	
PSPH	phosphoserine phosphatase	protein coding	Deficiency of this protein is thought to be linked to Williams syndrome.
AK023809	None	None	None
SRP14-AS1	SRP14 divergent transcript	ncRNA	
RP11-87F15.2	None	None	None
AB075489	None	None	None
KIAA0146	scaffold protein involved in DNA repair	protein coding	Involved in several processes, including cellular response to camptothecin; cellular response to hydroxyurea; and regulation of double-strand break repair.

### Analysis of m7G genes in lncRNAs of MX2/ HL60 cells by KEGG and GO enrichment

The biological classifications of m7G genes in lncRNAs of MX2/ HL60 cells were investigated by functional and pathway enrichment analyses.

**Fig. 4:**
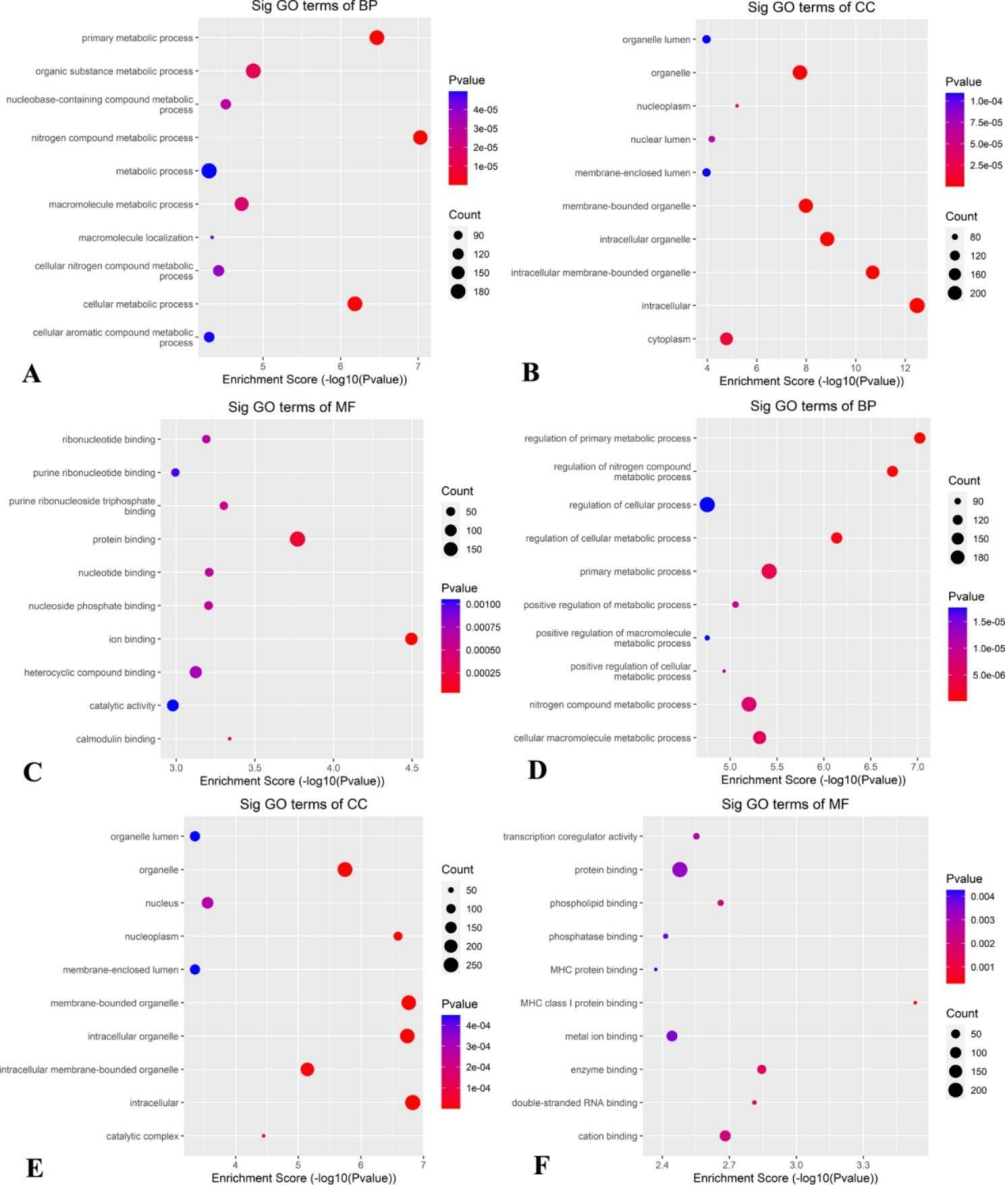
GO analysis of m7G genes in lncRNAs of HL60/MX2 cells. (**A-C**) The leading ten significantly enriched GO terms for (A) BPs, (B) CCs, and (C) MFs in up-methylated m7G genes of HL60/MX2 cells. (**D–E**) The leading ten significantly enriched GO terms of (D) BPs, (E) CCs, and (**F**) MFs in down-methylated m7G genes of MX2/ HL60 cells

The GO analysis revealed that BPs of m7G genes that were up-methylated in MX2/ HL60 cells were significantly enriched in cellular metabolic process, nitrogen compound metabolic process, organic substance metabolic process, and primary metabolic process, while the genes with m7G that were down-methylated were mainly related to nitrogen compound metabolic process and primary metabolic process, as well as their regulation. Enrichment of MF of the up-methylated m7G genes in MX2/ HL60 cells was basically in protein binding, ion binding, and heterocyclic compound binding, while the genes with m7G that were down-methylated were mainly related to metal ion binding, cation binding, enzyme binding, and protein binding. Enrichment of CC of the up-methylated m7G genes in MX2/ HL60 cells was basically in cytoplasm, intracellular, intracellular membrane-bounded organelles, intracellular organelles, membrane-bounded organelles, and organelles, which is consistent with methylation sites upregulated in HL60 cells (Fig. 4).

**Fig. 5:**
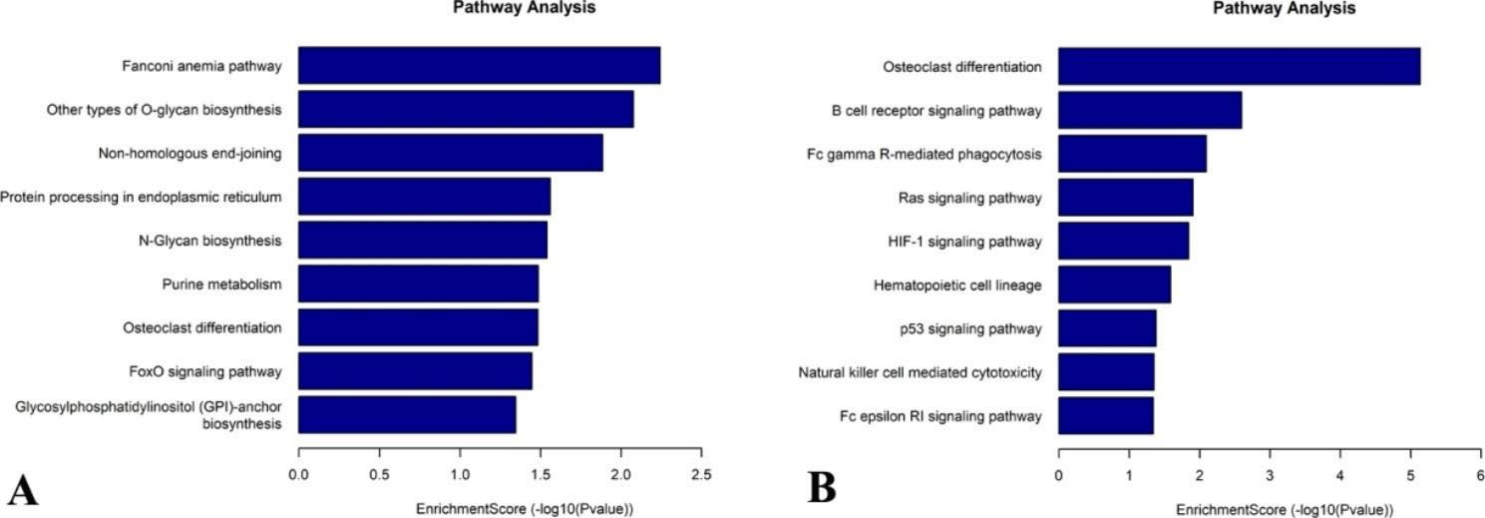
KEGG analysis of m7G genes in lncRNAs of MX2/ HL60 cells. (**A**) Top 10 significantly enriched pathways for up-methylated m7g genes in MX2/ HL60 cells. (**B**) Top 10 significantly enriched pathways for down-methylated m7G genes in MX2/ HL60 cells

The KEGG pathway analysis [23–25] revealed significant enrichment of lncRNAs with up-methylated m7G in HL60-MX2 cells primarily in the fanconi anemia pathway, other types of O-glycan biosynthesis, and non-homologous end-joining. Conversely, lncRNAs with down-methylated m7G showed significant enrichment in the Fc gamma R-mediated phagocytosis, B cell receptor signaling pathway, and osteoclast differentiation (Fig. 5).

## Discussion

Risk stratification in AML patients was conducted based on the results of cytogenetic and molecular analyses [27]. Specifically, the AML cases can be categorized into three sub-groups: favorable, intermediate, and poor outcomes. In the favorable sub-group, inv(16), t(8;21) and t(15;17) are most common cytogenetic abnormalities, while those with adverse prognosis exhibited abnormal 3q and 11q23 abnormalities besides t(9;11), as well as complex karyotypes. In the intermediate sub-group, most patients exhibit normal karyotypes and this case is regarded as CN-AML (cytogenetically normal AML). CN-AML accounts for 50% of adult AML patients, and it refers to patients with significantly broad diversity of clinical outcomes and molecular characterization. Meanwhile, CN-AML patients exhibited a wide range of molecular features and a wide diversity of clinical outcomes. Despite the FDA approval of several medicine for the treatment of AML, primary and secondary drug resistances remain a major issue in recent years. In one specific sub-group, some patients exhibited complete remission, while others showed drug resistance. Although the mechanisms of AML resistance to multiple drugs have been thorough investigated (genetic biomarkers), it is increasingly difficult for a single target to achieve AML treatment. The current AML classification faces huge challenges in prognosis and effective treatment due to the heterogeneity in each subgroup [28–30].

Epigenetic alterations, especially methylation, have emerged as effective molecular markers for evaluation of prognosis or drug therapeutic potential of cancers. Genes methylation has a high incidence (60-90%) in gastric cancer, Hodgkin, non-Hodgkin’s lymphoma, and breast cancer [31–34]. Located in cytoplasm and nuclei, eIF4E plays a key role in export and translation of specific m(7)G-capped mRNAs, respectively [35]. Nuclear accumulation of eIF4E in AML patients is related to increased export of transcripts, which are responsible for encoding oncoproteins, dependent on eIF4E. Additionally, its subcellular localization is directly related to responses of the patient. However, the expression level of m7G, as well as its role in AML resistance, remain poorly clarified.

As a potential critical layer of biological regulation, lncRNAs are aberrantly expressed in AML by transcription factors, DNA methylation or histone modifications, as well as by post-transcriptional modifications. Dysregulated lncRNAs may alter activity and expression of mRNAs, proteins and miRNAs, leading to epigenetic alterations in AML. For instance, AC026150.8, which is upregulated in AML, is correlated with poor prognosis, and its overexpression is positively related to drug resistance of AML cells, and confirms its scaffolding effect combined with splicing factors [36]. WT1 was upregulated in HOX transcript antisense RNA by targeting miR-20a-5p, and the regulation of resistance of Adriamycin by curcumin in AML cells can be reversed by inhibiting miR-20a-5p [37]. The transcriptional expression of lncRNAs in AML has been extensively investigated. Nevertheless, the mechanism by which m7G methylation in lncRNAs promotes the progression of AML drug resistance remains unclear, and further exploration is needed. In our study, the extent of cell methylation was estimated by examining the ratio of m7G in AML cells and AML-resistant cells. RNA MeRIP-seq was employed to globally locate m7G in human AML resistant and non-resistant cells; the distribution and prevalence of m7G in both cells and corresponding cellular compartments were compared with each other to clarify the m7G methylation levels of lncRNA in AML resistant cells. A substantial increase of m7G modification in AML resistant cells compared to non-resistant cells was observed. Meanwhile, 764 lncRNAs were upregulated, with high expression levels in HL60/MX2 cells. On the other hand, 870 lncRNAs were downregulated in HL 60/MX2 cells. The results suggested a correlation of molecular heterogeneous genomic events in AML with drug resistance. Specifically, the m7G site and the lncRNA set of genes localized by the m7G site in the AML-resistant cells far exceeded those in the AML cells. This was observed in all chromosomes. Cluster analysis showed that the methylation level is an effective index to distinguish AML cells from drug-resistant cells, confirming the correlation of m7G with AML resistance.

The present study utilized RNA MeRIP-seq to investigate m7G methylation over the transcriptome in human AML-resistant cells. The analysis revealed a total of 7,049 m7G peaks in HL-60 cells and 6,170 m7G peaks in HL60/MX2 cells. These peaks were associated with annotated genes, with 6,491 genes mapped in HL-60 cells and 5,757 genes mapped in HL60/MX2 cells. The study identified specific m7G motifs in the two cell types. In HL-60 cells, the most conservative motif was determined to be URUUUY (R = A/G, Y = U/C), while in HL60/MX2 cells, the most conservative motif was YMCAG (Y = C/U, M = C/A). The presence of these motifs indicated significant differences in the sequences of m7G methylation between the two cell groups. The density of m7G peaks in lncRNAs was also analyzed based on the cell type. The results showed that approximately 50% of lncRNAs in HL-60 cells and 45.9% of lncRNAs in HL60/MX2 cells contained m7G peaks in the CDS region. The percentages varied in other regions, such as 3’UTR, 5’UTR, stop codon (stopC), and start codon (startC), suggesting differential distribution patterns of m7G methylation in lncRNAs between the two cell types. Furthermore, the study analyzed the sources of methylated lncRNAs and found that most of the methylated lncRNAs were derived from exon sense overlapping regions, followed by intergenic regions. The distributions of lncRNA methylation sites across chromosomes were also examined, revealing differences between the two groups. The X chromosome and chromosome 2 exhibited notable differences in the distribution of lncRNA methylation sites. The differential expression of lncRNAs based on m7G methylation was assessed, and it was observed that 301 lncRNAs were upregulated and 355 lncRNAs were downregulated in HL60/MX2 cells compared to HL-60 cells. The study also provided a list of the top 10 upregulated and downregulated lncRNAs. Functional and pathway enrichment analyses were performed to investigate the biological classifications of m7G genes in lncRNAs of HL60/MX2 cells. GO analysis revealed enriched BPs and MFs that were associated with the up-methylated and down-methylated m7G genes. The up-methylated genes were enriched in cellular metabolic processes, protein binding, and ion binding, while the down-methylated genes were related to nitrogen compound metabolic processes and metal ion binding, among others. The KEGG pathway analysis demonstrated that the lncRNAs with up-methylated m7G in HL60/MX2 cells were enriched in pathways, such as the fanconi anemia pathway, other types of O-glycan biosynthesis, and non-homologous end-joining. On the other hand, the lncRNAs with down-methylated m7G were enriched in pathways including Fc gamma R-mediated phagocytosis, B cell receptor signaling pathway, and osteoclast differentiation.

Uptake of amino acid, catabolism and homeostate levels are elevated in LSCs (leukemia stem cells). LSCs that are obtained from naive AML patients are dependent on the metabolism of amino acids for OP (oxidative phosphorylation) and survival [35]. LSCs isolated from relapsed AML patients are independent of metabolism of amino acids owing to compensation by increased metabolism of fatty acids (FAs). It is known that cancer cells primarily rely on glycolysis for energy production rather than oxidative phosphorylation (OP). However, recent studies have demonstrated that cancer stem cells (CSCs) in different types of tumors depend on oxidative phosphorylation and display lower glycolytic reserves compared to non-stem cancer cells. The metabolic shift known as the Warburg effect, characterized by aerobic glycolysis, plays a pivotal role in cancer cells, leading to increased lactate production and heightened glucose consumption [35]. Metabolic plasticity and reprogramming are correlated with drug resistance, metastasis, and tumorigenesis [38]. For instance, mutations of Flt3-ITD elicit AKT-mediated PHOS of mitochondrial HK2 (hexokinase 2) and facilitate aerobic glycolysis in AML cells. 3-Brop (3-Bromopyruvate) leads to increased cell necrosis by sensitizing tumor cells to therapeutic performances of doxorubicin and sorafenib. mTORC1 is constitutively activated in AML cells and plays a significant role in the sensitivity of glycolytic inhibitors. The biosynthesis of FAs can be generated by reprogramming of glycolysis and glutaminolysis, thus facilitating survival and growth of cancer cells. In summary, the metabolic reprogramming signatures of AML were observed, and the inhibition of related pathways may contribute to the sensitivity of chemotherapeutic medicine. Interestingly, our Subsequent biological function predictions revealed that lncRNAs upregulated by m7G modification methylation were mainly enriched in complement activation and glycolysis/gluconeogenesis-related metabolic pathways, which may suggest that in AML cells, regulation of lncRNAs by m7G methylation could positively regulate glycolysis/gluconeogenesis-related metabolic pathways leading to cell drug resistance.

Additionally, it was noted that certain genes, which exhibited low expression levels in AML cells, demonstrated high expression in AML-resistant cells accompanied by the elevated levels of methylation. However, the precise role of this methylation in AML resistance necessitates further investigation. The present study provided a new perspective for the future study on the role of m7G methylation in drug resistance of AML cells to discover new therapeutic targets.

## Conclusions

In conclusion, the present study explored the regulatory mechanisms of m7G methylation in AML resistance. The findings shed light on the identification of differentially methylated lncRNAs, specific m7G motifs, and their distribution patterns in AML-resistant cells compared with AML cells. The results suggest a potential role for m7G methylation in modulating gene expression in AML-resistant cells. However, it is important to note that further investigation is warranted to fully understand the epigenetic alterations associated with AML resistance. Future research in this area may provide valuable insights into the underlying mechanisms and potential therapeutic targets for overcoming AML resistance.

## Data Availability

The datasets presented in this study can be found in online repositories. The names of the repository/repositories and accession number(s) can be found in the following link: https://www.ncbi.nlm.nih.gov/geo/query/acc.cgi?acc=GSE201096.
